# H3K18 lactylation of senescent microglia potentiates brain aging and Alzheimer's disease through the NFκB signaling pathway

**DOI:** 10.1186/s12974-023-02879-7

**Published:** 2023-09-11

**Authors:** Lin Wei, Xiaowen Yang, Jie Wang, Zhixiao Wang, Qiguang Wang, Yan Ding, Aiqing Yu

**Affiliations:** 1 Department of Clinical Laboratory, Hunan Provincial People’s Hospital, Central Laboratory of Hunan Provincial People’s Hospital, The First-Affiliated Hospital of Hunan Normal University, Changsha, 410000 China; 2 Hubei Key Laboratory of Embryonic Stem Cell Research, Department of Laboratory Medicine, Hubei University of Medicine, Taihe Hospital, The Affiliated Hospital of Hubei University of Medicine, Shiyan, 442000 China

**Keywords:** Senescent microglia, H3K18 lactylation, NFκB, Brain aging, Alzheimer’s disease

## Abstract

Cellular senescence serves as a fundamental and underlying activity that drives the aging process, and it is intricately associated with numerous age-related diseases, including Alzheimer's disease (AD), a neurodegenerative aging-related disorder characterized by progressive cognitive impairment. Although increasing evidence suggests that senescent microglia play a role in the pathogenesis of AD, their exact role remains unclear. In this study, we quantified the levels of lactic acid in senescent microglia, and hippocampus tissues of naturally aged mice and AD mice models (FAD^4T^ and APP/PS1). We found lactic acid levels were significantly elevated in these cells and tissues compared to their corresponding counterparts, which increased the level of pan histone lysine lactylation (Kla). We aslo identified all histone Kla sites in senescent microglia, and found that both the H3K18 lactylation (H3K18la) and Pan-Kla were significantly up-regulated in senescent microglia and hippocampus tissues of naturally aged mice and AD modeling mice. We demonstrated that enhanced H3K18la directly stimulates the NFκB signaling pathway by increasing binding to the promoter of Rela (p65) and NFκB1(p50), thereby upregulating senescence-associated secretory phenotype (SASP) components IL-6 and IL-8. Our study provides novel insights into the physiological function of Kla and the epigenetic regulatory mechanism that regulates brain aging and AD. Specifically, we have identified the H3K18la/NFκB axis as a critical player in this process by modulating IL-6 and IL-8. Targeting this axis may be a potential therapeutic strategy for delaying aging and AD by blunting SASP.

**Supplementary Information:**

The online version contains supplementary material available at 10.1186/s12974-023-02879-7.

## Introduction

Cellular senescence is a complex process involving factors that lead to a decline in cell growth and proliferation, ultimately resulting in the irreversible arrest of the cell cycle. This includes replicative senescence (caused by replicative exhaustion), and premature senescence (caused by oncogene activation or chemotherapeutics treatment) [1]. Cellular senescence is the foundation and premise of tissue, organ, and even organismal aging [2, 3]. In addition, the expression of functional proteins, and the synthesis and release of cytokines are abnormal in senescent cells, which leads to the functional decline of tissues and organs [4]. Studies show that cellular senescence is the main pathogenic mechanism and a potential therapeutic target of brain aging and neurodegeneration diseases, such as Alzheimer's disease (AD) [5, 6], Parkinson's disease (PD) [7, 8] and osteoarthritis (OA) [9–12], etc. However, the role and mechanism of cellular senescence in aging and aging-related diseases require further research.

AD is a neurodegenerative disease associated with aging, characterized by progressive cognitive impairment. The deposition of amyloid-β (Aβ) plaques and tau neurofibrils are two typical pathological features of AD, which are the main events and causes of neuronal loss, ultimately leading to cognitive decline in AD [13, 14]. As the global population ages, the number of AD patients is increasing. Currently, the number of AD patients is over 40 million, and it is expected to exceed 100 million by 2050 [8]. Studies have shown that postmenopausal older women are at a higher risk of developing AD [15, 16].This increase in the number of AD patients will create enormous pressure and burden for patients, their families, and society as a whole [15]. Unfortunately, there is no effective therapy for AD, and the pathogenesis of AD needs to be further investigated.

The microglia are a group of immune cells that reside in the brain, involved in several biological processes, including the pruning of synapses, the formation of myelin sheaths, and the removal of cellular debris and misfolded proteins, which are crucial for brain development, maintenance of brain physiological function and occurrence and development of various brain diseases [17–19]. Although there is increasing evidence that microglia play a role in the pathogenesis of AD, the potential molecular mechanisms remain unclear. In particular, neuroinflammation in AD is receiving increasing attention, and microglia play a role in several aspects of neuroinflammation [20]. Reactive Gliosis (i.e., the microglia response to nerve tissue damage, pathogen infection, or other injuries) has been observed to co-locate closely with Aβ plaques in animal models of AD and postmortem brain tissue of AD patients. This suggests that microglia neuroinflammation may be an early event in AD, and that microglia pro-inflammatory activation is a hallmark of AD [13, 21]. In addition, microglia can undergo a metabolic switch from oxidative phosphorylation (OXPHOS) to aerobic glycolysis in response to stress stimuli [22, 23]. Importantly, lactic acid, a product derived from aerobic glycolysis, was found to directly promotes the release of microglia proinflammatory cytokines, such as tumor necrosis factor (TNF-α), interleukin (IL-6), and interleukin 1B (IL-1β) [24], but the underlying mechanisms are unclear. Metabolically active senescent microglia-mediated neuroinflammatory responses (e.g., via SASP) are thought to further threaten senescent neurons, thus driving the progression of age-related neurodegeneration [25], such as AD. However, the effect of senescent microglia on AD and the underlying mechanisms are not well-understood.

In eukaryotic cells, protein post-translational modification (PTM) enables rapid functional adaptation to various intracellular and extracellular signals by regulating enzyme activity and protein stability [26]. PTM disorders can lead to a variety of pathological conditions, such as defective sperm development [27], malignant transformation [28–30] and depression [31]. With the application of modern mass spectrometry (MS), a series of novel short-chain lysine acylations have been identified, such as lactylation [32], crotonylation [33], β-hydroxy isobutyrylation [34] and succinylation [35]. Although these novel acylation has a structural parallel with the well-known acetylation, their physical function are distinctive. Histone Lysine lactylation (Kla) is a novel epigenetic modification that can affect gene expression [36]. Interestingly, lactate, which is produced by glycolysis under hypoxic conditions or during the bacterial challenge, stimulating histone lactylation and subsequently activating downstream gene expression [32]. In addition, histone lactylation plays an important role as an epigenetic regulator in pathogenesis. For example, elevated histone Kla at the gene sites of reparative macrophages can promote the transition of macrophages from an inflammatory state to a repair state in response to microbial ligands and various deleterious signals [37]. Glis1-induced histone Kla at the pluripotency gene locus promotes somatic reprogramming [38]. Histone lactylation drives tumorigenesis by regulating different downstream targets or signaling, such as m6A reader protein YTHDF2 in ocular melanoma [30], TGF-β in hepatocellular carcinoma [39], and METTL3-mediated RNA m6A in tumor-infiltrating myeloid cells [40]. Furthermore, it has been found that nerve excitation can induce the lactic modification of brain proteins and that lysine lactate in brain cells is regulated by systemic changes in lactate levels, nerve excitation and behavior-related stimuli [41]. Recently, Wang et al. found that histone lactylation can promote reparative gene activation post myocardial infarction [42]. Collectively, these studies indicate that histone Kla is an important molecular mediating the function of lactic acid, and it is involved in various pathophysiologies as mentioned above. However, there are few reports on the study of histone Kla in aging and AD.

In this study, we found that elevated lactic acid levels promote H3K18 lactylation in senescent microglia and the hippocampus of naturally aged mice and AD mice. Treatment of senescent microglia with Lactate dehydrogenase inhibitors leads to a dramatic decrease in lactic acid levels, which cause a significant decrease in histone Kla. We identified all histone Kla sites in senescent microglia, and found that H3K18la was significantly upregulated in senescent microglia, and the hippocampus of naturally aged mice and AD mice. We demonstrate that enhanced H3K18la directly stimulates the NFκB signaling to promote SASP, thereby facilitating aging and AD phenotypes.

## Experimental section

### Animal care and ethics statement

Eight-week-old C57BL/6 mice were purchased from the animal Centre of Hubei University of Medicine. APP/PS1 and FAD^4T^ AD modeling mice were purchased from SPF (Beijing) Biotechnology Co. Ltd. and GemPharmatech Co. Ltd., respectively. All the mice were housed in a temperature- and light-controlled specific pathogen-free (SPF) animal facility and allowed free access to food and water. The mice that were fed on a normal diet for at least 24 months were considered to be naturally aged mice.

### Cell culture

BV_2_ cells were cultured in Dulbecco's Modified Eagle Medium (DMEM) supplemented with 10% fetal bovine serum. HMC3 cells were cultured in Minimum Essential Medium (MEM) containing Non-Essential Amino Acids (NEAA) supplemented with 10% fetal bovine serum. Cells were passaged every 3–4 days. The cells were maintained in a constant temperature incubator at 37 ℃ and 5% carbon dioxide.

### Histone extraction

Histones acid extraction from tissues and cells was performed as previously described [43]. The collected tissues or cells were resuspended in lysis buffer containing protease inhibitors (10 mM Tris–HCl, 1 mM KCl, 1.5 mM MgCI_2_, 1 mM DTT, 1.5 mM H_2_SO_4_) and was shaken overnight at 4 ℃ to extract nuclear proteins. The supernatants were then collected after centrifugation at 16,000*g* for 10 min at 4 ℃, and histones were precipitated on ice using a final concentration of 33% trichloroacetic acid. The histones were washed, dried and dissolved in H_2_O, and a protein loading buffer was added. Western blotting was performed after 10 min in a metal bath at 98 ℃.

### Doxorubicin induces microglia senescence

To establish a cell model of senescence, microglia were treated with doxorubicin as previously reported [44]. When the cells reached a confluency of approximately 70%, they were exposed to complete medium containing doxorubicin (920 nM for BV2 and 230 nM for HMC3 final concentration of 230 nM) for 24 h. The old medium was removed and cells were washed with PBS to eliminate the effect of the drug. A fresh complete medium was added and incubated for 48 h. SA-β-Gal staining and Western blotting were performed to verify the successful induction of senescence in the cells.

### Measurement of lactate levels

The cells were harvested and transferred to centrifuge tubes, and then washed with pre-cooled PBS. The supernatant was removed after centrifugation, and 1 mL of lactate assay buffer was added. Cells were sonicated by ultrasound in an ice bath for 5 min (200 W, 3 s, 7 s intervals, 30 times). After centrifugation at 12,000*g* for 5 min at 4 ℃, the supernatant was transferred to a new EP tube. Frozen hippocampal tissue was mixed with 1 ml lactate assay buffer and homogenized on ice bath, centrifuged at 12,000*g* for 5 min at 4 ℃. The supernatant was collected for determination of lactate levels using the CheKine™ micro-Lactate Assay Kit (KTB1100).

#### Immunofluorescence staining

Cells were seeded on coverslips (Biosharp) in 12-well plates. Following the induction of cell senescence, the culture medium was removed and the cells were washed thrice with PBS. The cells were fixed with 4% paraformaldehyde for 10 min at room temperature and permeated with 0.1% Triton X-100 for 10 min. This was followed by blocking with 5% BSA for 30 min, incubation with the corresponding primary antibodies overnight at 4 ℃. The cells were then incubated with corresponding fluorescent secondary antibody for 1 h at room temperature in the dark, and the DAPI staining reagent was stained for 10 min in the dark. Finally, the coverslips were placed onto slides using an anti-fluorescence quenched sealer. Images were captured with an Olympus BX53 + DP74 forward fluorescence microscope.

### Clonal expansion assay

The clonal expansion assay was performed as described previously [45]. In briefly, cells in the dish were digested into individual cells by incubation with pancreatic enzymes. Approximately 5000 to 8000 cells were seeded into each well of 6-well plates. Senescence was induced by treatment with doxorubicin after about 7 days of culture. Cells were washed twice with PBS, fixed with methanol for 30 min at room temperature, stained with crystal violet for 30 min at room temperature, and rinsed with tap water.

### Senescence-associated beta-galactosidase (SA-β-gal) staining

SA-β-gal activity was performed as reported previously [46]. Concisely, cells were washed once with PBS and fixed with β-galactosidase staining solution for 15 min at room temperature. Cells were washed with PBS 3 times for 3 min each time. Cells were treated with staining solution (prepared according to C0602 instructions) and sealed with sealing film. After overnight incubation in the 37 ℃, the cells were imaged using an Olympus IX53 + DP73 inverted microscope.

### Real-time label-free dynamic cell analysis

A complete medium was added to the E-plate and the background impedance values were detected. Cells in the logarithmic growth phase were digested with trypsin and counted. About 3000 cells were seeded into each well and placed on a test table in a cell incubator. Finally, real-time detection of dynamic cell proliferation was performed.

### Multiplexed immunohistochemistry (mIHC)

mIHC was performed as described previously [47]. Briefly, fresh hippocampal tissues were fixed by 4% paraformaldehyde overnight, embedded in paraffin and then cut into slices. After dewaxing and hydration, the slides were immersed in antigen retrieval solution in a microwave to restore antigen. Next, the slides were blocked with 10% serum at room temperature for 10 min with shock. After removing blocking fluid from the slides, a diluted anti-Iba1 (ab178846, Abcam, USA) or pan-Kla (PTM-1401, PTM BIO, China) primary solution was added to the sample area. After incubation at room temperature for 1 h with humectant shock, the slides were washed with 1 × TBST buffer for 3 min and repeated once. First, a signal amplification reaction solution was added to a slide. Then, the sample area was immersed in the solution to amplify the fluorescence staining signal. After incubating for 10 min at room temperature, the slides were soaked in 1 × TBST buffer for 3 min at room temperature, and this process was repeated three times. Subsequently, a new primary antibody staining was repeated until all primary antibodies were stained. Finally, the DAPI working fluid was added to the slides and incubated at room temperature. Next, 1 × TBST buffer was used to soak the slides for 3 min at room temperature, and repeated 3 times. The stained sections were observed and imaged under fluorescence microscope.

### Protein extraction and western blotting

Tissues or cells were lysed with RIPA lysis buffer containing protease and phosphatase inhibitors for 30 min on ice, centrifuged at 12,000 rpm for 15 min at 4 ℃ and supernatant collected. BCA method was used to. The protein concentration was determined by the BSA method and the final concentration was adjusted to ensure consistence. The protein samples were mixed with protein loading buffer, and boiled at 98 ℃ for 10 min. Next 30 μg of protein was separated by 12% SDS–PAGE. After electrophoresis, the proteins were transferred onto polyvinylidene difluoride membranes (0.45 μM pore size). The membranes were blocked with TBST buffer containing 5% skim milk powder for 2 h at room temperature, then incubated with the corresponding primary antibodies overnight at 4 ℃ on the shaker. Membranes were washed three times with TBST buffer and then conjugated with a horseradish peroxidase labeled secondary antibody for 2 h at room temperature. Protein bands were visualized using the ChemiDoc Acquisition Image XRS + system (Bio-Rad Laboratories). The following primary antibodies were used: l-Lactyl Lysine (1:1000 dilution, PTM-1401RM, PTM BIO), H4K12la (1:1000 dilution, PTM-1411RM, PTM BIO), H4K8la (1:1000 dilution, PTM-1415, PTM BIO), H4 (1:1000 dilution, PTM-1009, PTM BIO), H3K18la (1:1000 dilution, PTM-1406RM, PTM BIO), H3K14la1:1000 dilution, (PTM-1414, PTM BIO), P21 (1:3000 dilution, ab109520, Abcam), P53 (1:3000 dilution, ab183544, Abcam), P16 (1:1000 dilution, A0262, ABclonal), H3 (1:1000 dilution, AF0009, Beyotime), GAPDH (1:5000 dilution, 60004-1-Ig, Proteintech), NF-kappaB p65 (1:3000 dilution, 8242T, Cell Signaling Technology), Phospho-NF-kappaB p65 (Ser536) (1:3000 dilution, 3033T, Cell Signaling Technology). We used ImageJ software to quantify the band intensities, and inserted the corresponding numbers below each band to represent the (average) ratio for the protein or modification relative to the loading control. This approach allowed us to accurately assess the levels of each protein or modification in our samples.

### Chip-seq and data analysis

BV2 cells were added into fresh medium in plates at a density of 90% (1 × 10^7^/well) and cultured in an incubator at 37 ℃ for 30 min. They were cross-linked with formaldehyde at a final concentration of 1% for 10 min at room temperature and with glycine at a final concentration of 125 mM for 5 min at room temperature. The cells were rinsed three times with pre-cooled PBS. Cell collection was performed through scraping and centrifugation at 4 °C to remove all supernatants. Cells frozen in liquid nitrogen were rapidly thawed on dry ice for subsequent experiments. To prepare the sequencing data, FastP (v0.20.1) was employed to trim adapter sequences and remove low-quality bases under default settings. To align the cleaned reads to the mouse genome (C57BL). Bowtie2 (version 2.4.2) was applied under default parameters. Next, model-based analysis was performed using Chip-Seq (MACS2) software (version 1.4.2) to identify enriched regions and to call peaks by comparing reads from the IP sample with the input sample after removal of potential PCR duplicates with SAMtools (version 1.11). The peak annotation was conducted using the R package ChIPSeeker. For data visualization, the Integrative Genomics Viewer (IGV) was utilized, with MACS2 software's conversion formats wig files being employed to generate the visual representation. Heatmaps or distinct histone marks were created using the deepTools 1.5.2 software.

### RNA extraction library construction and sequencing

Total RNA was extracted, and mRNA was purified from total RNA (5 µg) using Dynabeads Oligo (dT) (Thermo Fisher, CA, USA) with two rounds of purification. The mRNA was fragmented into short fragments using divalent cations at high temperature. The cleaved RNA fragments were reverse-transcribed to form cDNA using a SuperScript™ II Reverse Transcriptase (Invitrogen, cat. 1896649, USA). The average insert size for the final cDNA libraries was 300 ± 50 bp. Finally, we performed the 2 × 150 bp paired-end sequencing (PE150) on an Illumina Novaseq™ 6000 (LC-Bio Technology CO., Ltd., Hangzhou, China) following the vendor's manufacturer’s instructions.

### Statistical analysis

The statistical analyses were analyzed by two-tailed unpaired Student’s t test using GraphPad Prism software unless otherwise indicated. All quantitative data from three independent biological repeats were presented as the means ± standard deviations (SDs). All experiments were performed as three independent biological replicates unless otherwise indicated. A *p* < 0.05 was considered statistically significant.

## Results

### Increased lactate levels in senescent microglia, and hippocampus tissues of naturally aged mice and AD mice

**Fig. 1:**
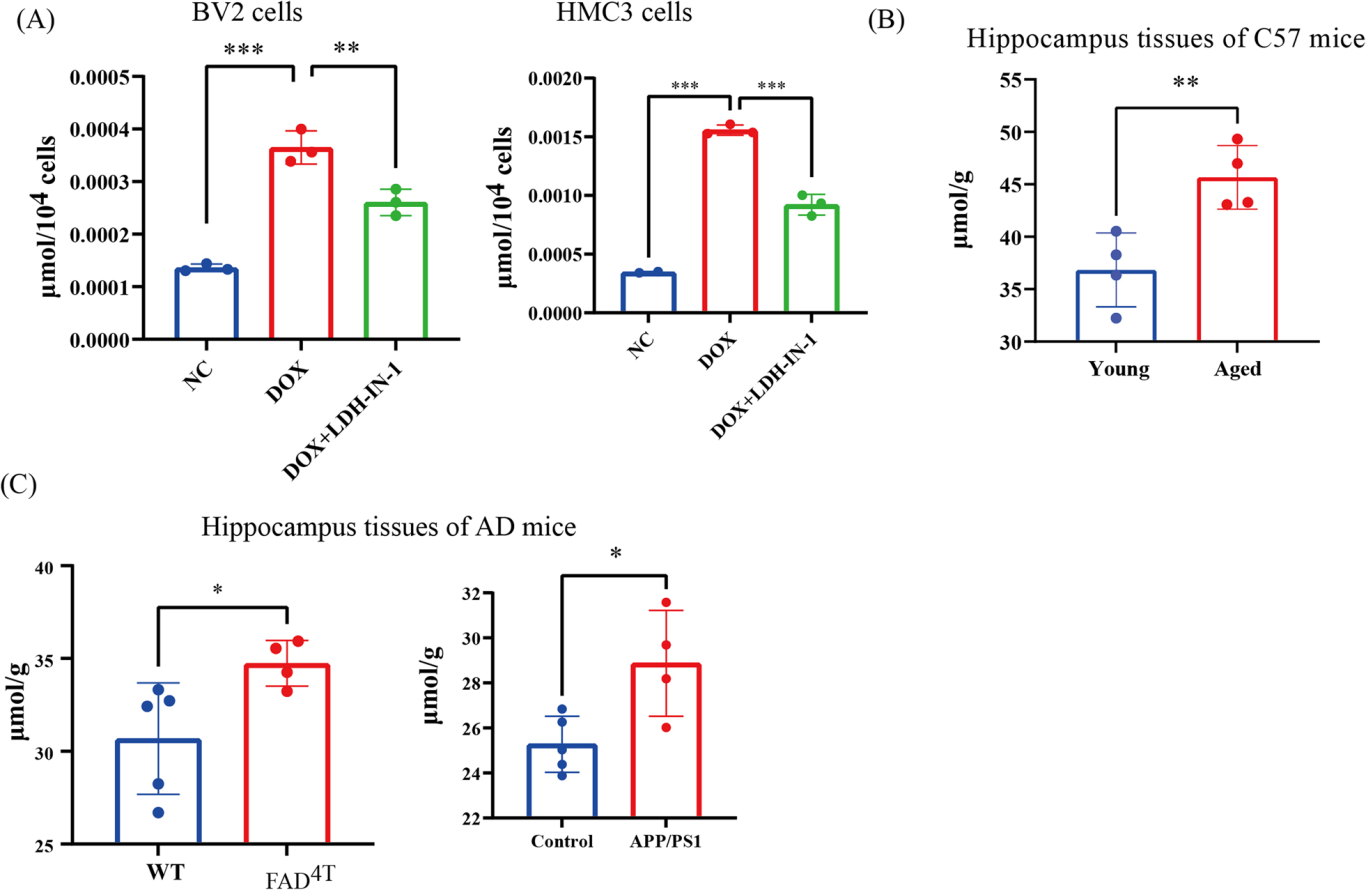
The concentration of lactate is elevated in senescent microglia and hippocampus tissues of naturally aged mice. **A** Lactate levels in proliferating and premature senescent microglia (BV2 and HMC3 cells), and in proliferating and premature senescent microglia treated with LDH-IN-1. **B** Lactate levels in hippocampus tissues of young and naturally aged mice (*n* = 3 mice per group). **C** Lactate levels in hippocampus tissues of FAD^4T^, APP/PS1 and respective counterparts (*n* = 3 mice per group). Each bar represents the mean ± s.d. for triplicate experiments, *p < 0.05, **p < 0.01, ***p < 0.001

In this study, we investigated the lactate levels in microglia, microglia are known to be metabolically flexible and adaptable various stimuli throughout their lifespan [48]. To evaluate the metabolic reprogramming of microglia from OXPHOS to glycolysis, we measured the lactate levels in doxorubicin(dox)-induced premature senescent microglia (termed as BV2_Dox and HMC3_Dox) (a well-established cellular senescent model) (Additional file 1: Fig. S1a–h), proliferating microglia (termed as BV2_NC and HMC3_NC), as well as in the hippocampus of naturally aged mice (24-month-old mice and older (geriatric age)) (Additional file 1: Fig. S1i, j) and AD mice (two well-established AD mice model). The colorimetric assay revealed that lactate levels were significantly elevated in premature microglia (Fig. 1A), and the hippocampus tissues of naturally aged mice (Fig. 1B), FAD^4T^ mice and APP/PS1 mice (Fig. 1C). These findings are consistent with the previous reports indicating higher lactate concentrations AD patients compared to healthy wild type (WT). Notably, lactate levels are markedly reduced in dox-induced senescent microglia following treatment with lactate dehydrogenase inhibitor (LDH-IN-1) (Fig. 1A), suggesting that the increased lactate levels in dox-induced senescent microglia originate from glycolysis products.

### Histone Pan-Kla is substantially elevated in senescent microglia and hippocampus tissues of naturally aged mice

**Fig. 2:**
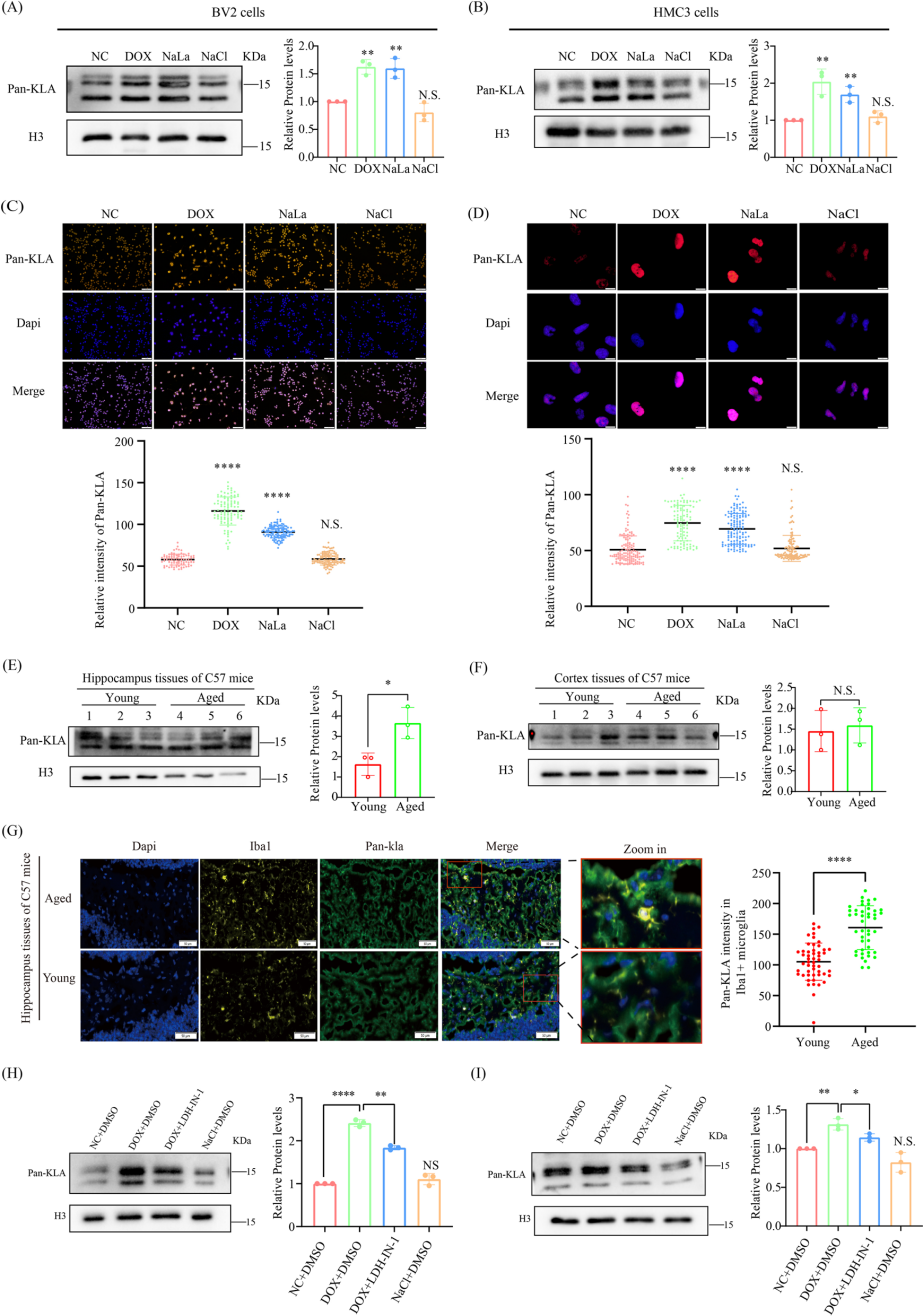
Histone Pan-Kla levels is increased in premature senescent microglia and hippocampus tissues of naturally aged mice. **A**, **B** Measurement of histone Pan-Kla levels in the indicated microglia groups by immunoblotting assay. **C**, **D** Detection of histone Pan-Kla levels in the indicated microglia groups by immunofluorescence. **E** Measurement of Pan-Kla levels in hippocampus tissues from young and naturally aged mice (*n* = 3 mice per group). **F** Multiplexed immunohistochemistry measurement of Pan-Kla levels in hippocampus tissues from young and naturally aged mice (*n* = 3 mice per group), scale bar, 50 μm. **G** Immunoblotting analyzes for Pan-Kla levels in the indicated microglia groups. Each bar represents the mean ± s.d. for triplicate experiments, *p < 0.05, **p < 0.01, ***p < 0.001, N.S.: no significance

As lactate or Sodium lactate can serve as a precursor metabolite to stimulate histone Kla [32], it prompted us to hypothesize that histone Kla may be altered in the context of aging. Therefore, Sodium lactate was used in this study to enhance lactylation after treatment of BV2 and HMC3 cells [32, 40]. Intriguingly, immunoblotting analysis of acid-extracted histones, cell immunofluorescence and multiple immunohistochemistry (mIHC) showed a significant increase in the levels of Pan-histone Kla in both senescent microglia (Fig. 2A–D) and hippocampus tissues of naturally aged mice (Fig. 2E), but not in the cortex tissues of naturally aged mice (Fig. 2F). In addition, mIHC revealed that the Pan Kla level is strikingly increased particularly around the microglia (Fig. 2G). Moreover, we found that the non-histone Pan Kla levels are also dramatically elevated in both senescent microglia, and hippocampus tissues of naturally aged mice, in relation to corresponding counterparts (Additional file 1: Fig. S2a, b), but no significant change was observed in the cortex (Additional file 1: Fig. S2c). Because the degree of increase in non-histone Pan Kla was less pronounced than that of histone Pan Kla. We focused our attention on histone Kla in the subsequent study. Similar to the alteration of lactate levels, elevated pan-histone Kla levels in dox-induced senescent microglia were significantly decreased when treated with lactate dehydrogenase inhibitor in BV2 (Fig. 2H) and HMC3 (Fig. 2I), indicating that the increased histone pan-Kla in senescent microglia indeed comes from elevated lactate concentrations.

### Global landscape of lactylome in BV2 cells

**Fig. 3:**
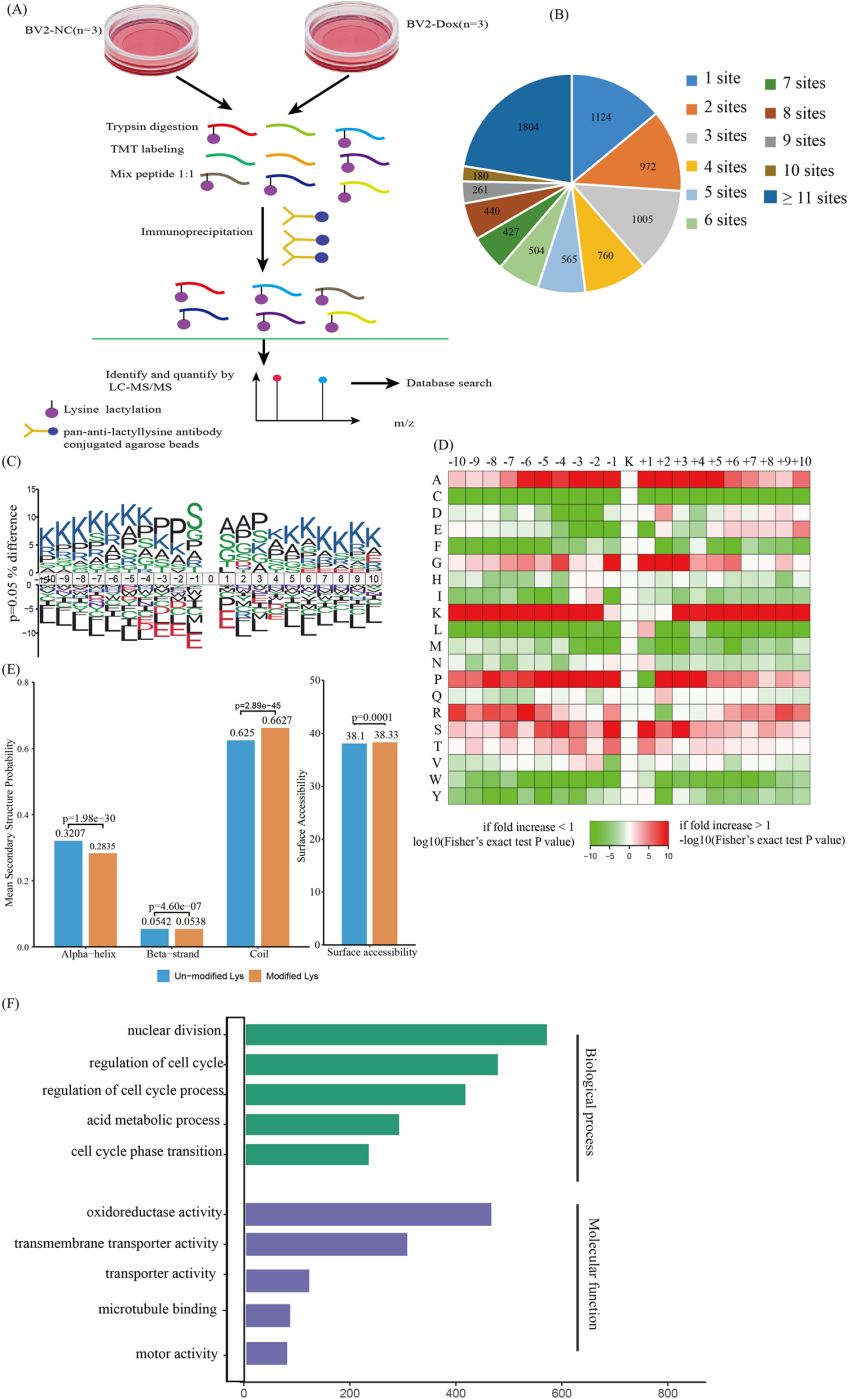
Identification of Kla proteome profile in BV2 cells. **A** Schematic representation of the experimental workflow of TMT labeling, affinity enrichment and mass spectrometry-based quantification of Kla in BV2_NC and BV2_Dox. **B** Pie chart displaying the distribution of the number of identified Kla sites per protein. **C** Motif analysis for the identified Kla proteins. **D** Icelogo representation showing flanking sequence preferences for all Kla sites between the upstream + 10 position and the downstream − 10 position. **E** Distribution of all lysines and lactylated lysines in the structured regions of proteins. **F** Bar graphs showing the representative ontology annotations enriched with Kla proteome

We have demonstrated that the lactate-derived histone pan-Kla level is increased in senescent microglia and hippocampus tissues of naturally aged mice. To gain a global view of the lactylome including histone and non-histone Kla, we used an integrated approach involving TMT (Tandem Mass Tags) labeling, HPLC (high-performance liquid chromatography) fractionation, affinity enrichment, and high-resolution LC–MS/MS (liquid chromatography–tandem MS) to investigate lactylome changes in dox-induced senescent BV2 cells (BV2_Dox) in relation to proliferative BV2 (BV2_NC). A total of 2 × 10^7^ cells for each TMT labeling were harvested and lysed, and then mixed with protein lysates in a 1:1 ratio. After trypsin digestion, tryptic peptides were labeled with their respective TMT reagent. After labeling, the sample was fractionated into fractions by high pH reverse-phase HPLC, and Kla-containing peptides were enriched with anti-Pan-Kla and analyzed by LC–MS/MS (Fig. 3A). A total of 60,159 Kla sites across 6715 were identified, with 57950 Kla sites from 6526 proteins quantified. Among these identified Kla proteins, 1124 (17.2%) had a single Kla site, 3112 (47.7%) had more than six Kla sites, and 1804 (27.7%) had more than ten Kla sites (Fig. 3B). Our study detected significantly more Kla sites, which hardly intersected with the previously reported lactylome data set [32].

We next analyze the amino acids flanking the identified Kla sites using iceLogo. Obvious enrichment of serine and alanine was found at − 1 and + 1 positions of Kla sites, respectively (Fig. 3C). Structural analysis of Kla proteins by NetSurfP software found that roughly 28.35% of Kla sites were situated in α helices, 5.38% were located in β strands, and the remaining 66.27% were found in irregular coils (Fig. 3D). The structural distribution model of Kla showed no apparent distinction compared with total protein lysine residues, indicating that there was no structural preference for Kla, at least in BV2 cells. The average surface structure accessibility of protein Kla was significantly (*p* < 0.001) less than that of total protein lysine residues, suggesting that Kla preferentially localizes within protein structures. (Fig. 3E). Analysis of Kla-modulated intracellular pathways by Gene Ontology (GO) disclosed that Kla proteins are participated in a variety of cellular life processes including cellular process, biological regulation, metabolic process, and response stimulus (Fig. 3F).

### Quantitative analysis of Kla proteome in BV2 cells

**Fig. 4:**
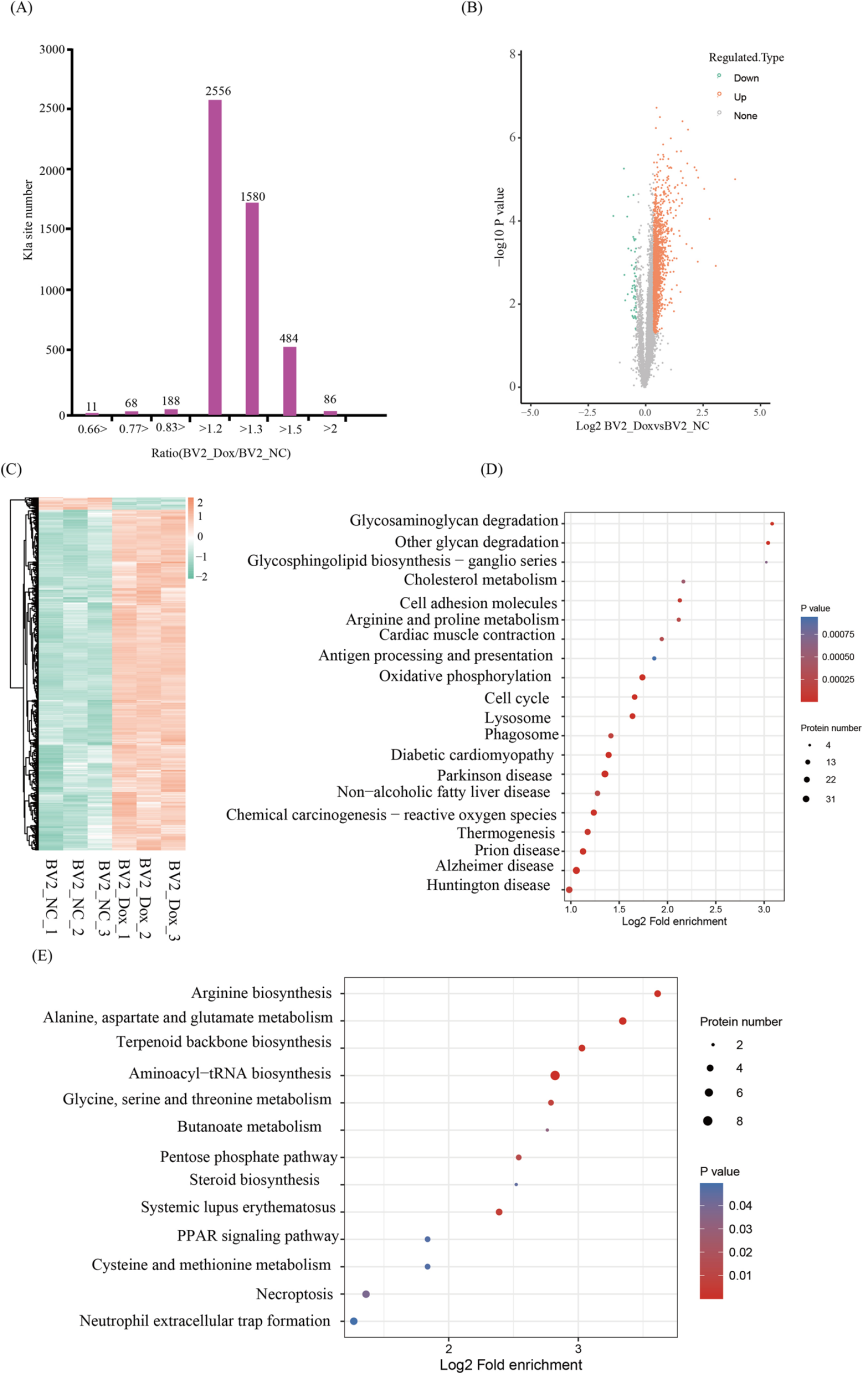
Quantification of lactylome in response to premature senescent BV2 cells. **A** Histogram showing the distribution of the relative ratio distribution of quantifiable Kla sites between BV2_Dox and BV2_NC. **B** Scatter plot showing changes in the quantifiable Kla sites between BV2_NC and BV2_dox. **C** Heat map showing the changes in the quantifiable Kla sites between BV2_NC and BV2_dox. **D**, **E** Bubble diagram showing KEGG pathway enriched with up-regulated Kla proteins (**D**) and down-regulated Kla proteins (**E**)

Subsequently, we quantified the changes in alteration of protein Kla relative to total protein abundance in BV2_Dox. The cutoff ratio for significant Kla changes between BV2_Dox and BV2_NC was set to be above 1.3 or below 0.77. The data revealed that 1580 Kla sites in 841 proteins were up-regulated, and 57 sites in 53 proteins were down regulated in BV2_Dox (b). KEGG (Kyoto Encyclopedia of Genes and Genomes) enrichment analysis found that up-regulated Kla proteins are mainly involved in oxidative phosphorylation, cell cycle, lysosome, phagosome, Parkinson, Diabetic cardiomyopathy, and Alzheimer disease (Fig. 4D). Meanwhile, down-regulated Kla proteins are enriched in pathways associated with Arginine biosynthesis, Alanine, aspartate and glutamate metabolism, Terpenoid backbone biosynthesis and Aminoacyl–tRNA biosynthesis (Fig. 4E).

### Elevated levels of H3K18la are apparent in premature senescent microglia and hippocampus tissues of naturally aged mice and AD disease modeling

**Fig. 5:**
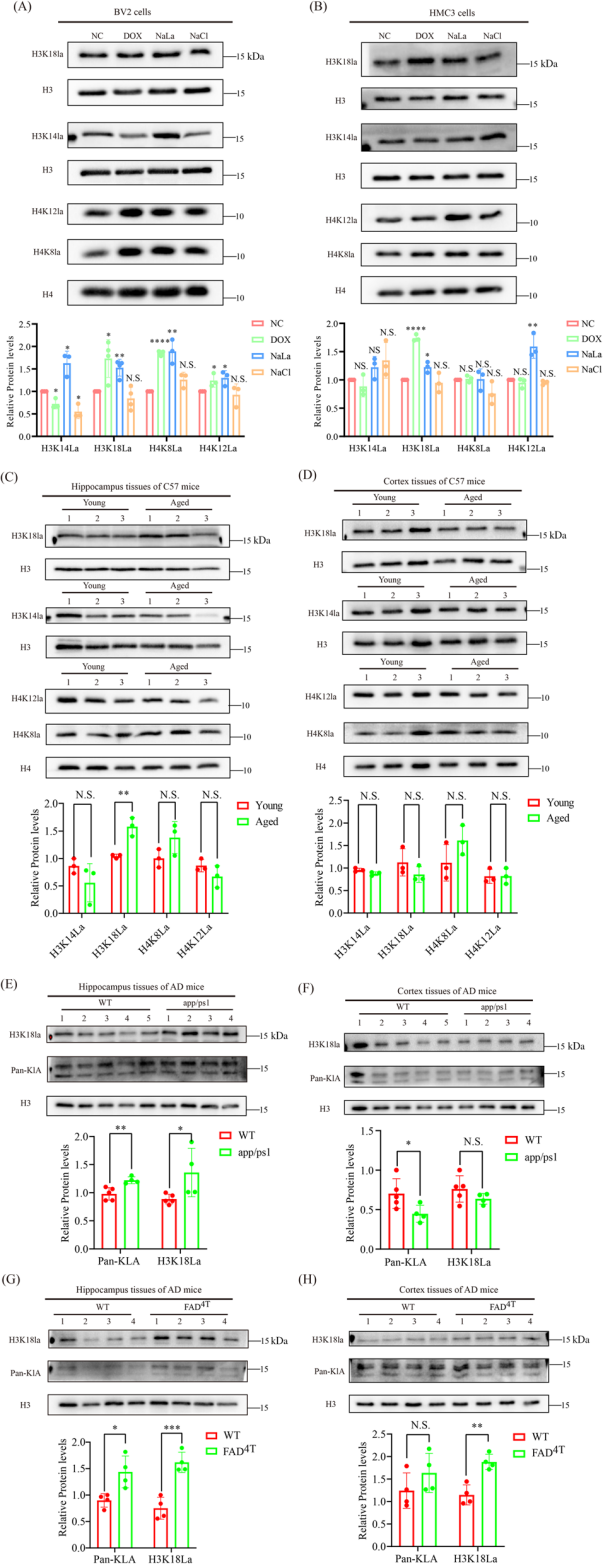
Validation of the MS-generated histone Kla sites. **A**, **B** Immunoblotting results showing the changes in levels of H3K14, H3k18, H4K8 and H4K12 Kla sites in the indicated BV2 (**A**) or HMC3 (**B**) cells using their corresponding site-specific Kla antibodies. **C** Immunoblotting evaluation of changes in Kla sites of H3K14, H3k18, H4K8 and H4K12 in hippocampus tissues from young and naturally aged mice with the corresponding site-specific Kla antibodies (*n* = 3 mice per group). **D**, **E** Immunoblotting results illustrating changes in Kla of H3K18, pan-Kla in hippocampus tissues of WT, and FAD4T and app/ps1 C57 mice (*n *= 3 mice per group in figure D, *n* = 5 WT mice and 4 app/ps1 mice in figure **E**. **F**–**H** Immunoblotting displaying changes in H3K18, pan-Kla in cortex of naturally aged C57 mice, FAD^4T^, app/ps1 C57 mice and corresponding counterparts (*n* = 5 WT mice and 4 app/ps1 mice in figure F, *n* = 4 mice per group in figure **G**, **H**). Each bar represents the mean ± s.d. for triplicate experiments, *p < 0.05, **p < 0.01, ***p < 0.001, N.S.: no significance

Histone Kla sites were identified by HPLC–MS/MS and the MS/MS spectra of histone Kla sites are shown (Additional file 1: Fig. S3a, b). We then compared our identified histone Kla sites in BV2 cells with histone Kla sites in mouse bone-marrow-derived macrophages (BMDMs) identified by Zhang et al. [33], and identified several overlapping histone Kla sites (Additional file 1: Fig. S3c). Next, we selected H4K8, H4K12, H3K14 and H3K18 to validate the MS-generated lactylome data. For this purpose, acid-extracted histones from proliferative microglia (BV2_NC, HMC3_NC), dox-induced senescent microglia (BV2_Dox, HMC3_Dox), and NacL or NaLa treated proliferative microglia were immunoprecipitated with corresponding histone pan-and sites-Kla antibody, and histone H3 antibody. The results showed that these histone sites were indeed lactylated in microglia and hippocampus tissues (Fig. 5A–C). Among these lactylated histone sites, only H3K18la is elevated in both dox-induced senescent microglia (Fig. 5A, B), hippocampus tissues of naturally aged mice (Fig. 5C) and AD model mice (Fig. 5D, E), which is in line with the previously reported results that H3K18la level is increased in hippocampus of AD mouse model compared to that of WT mice. Intriguingly, H3K18la level is not significantly altered in the cortex of naturally aged mice (Fig. 5F) and AD modeling mice (Fig. 5G, H), suggesting that H3K18la may be an important potential target involved in regulating aging and AD by functioning in the hippocampus region. Consequently, we focus our attention on the biological function of H3K18la implicated in aging and AD.

### Genome-wide analysis of the transcriptional consequences of H3K18la in senescent BV2 cells

**Fig. 6:**
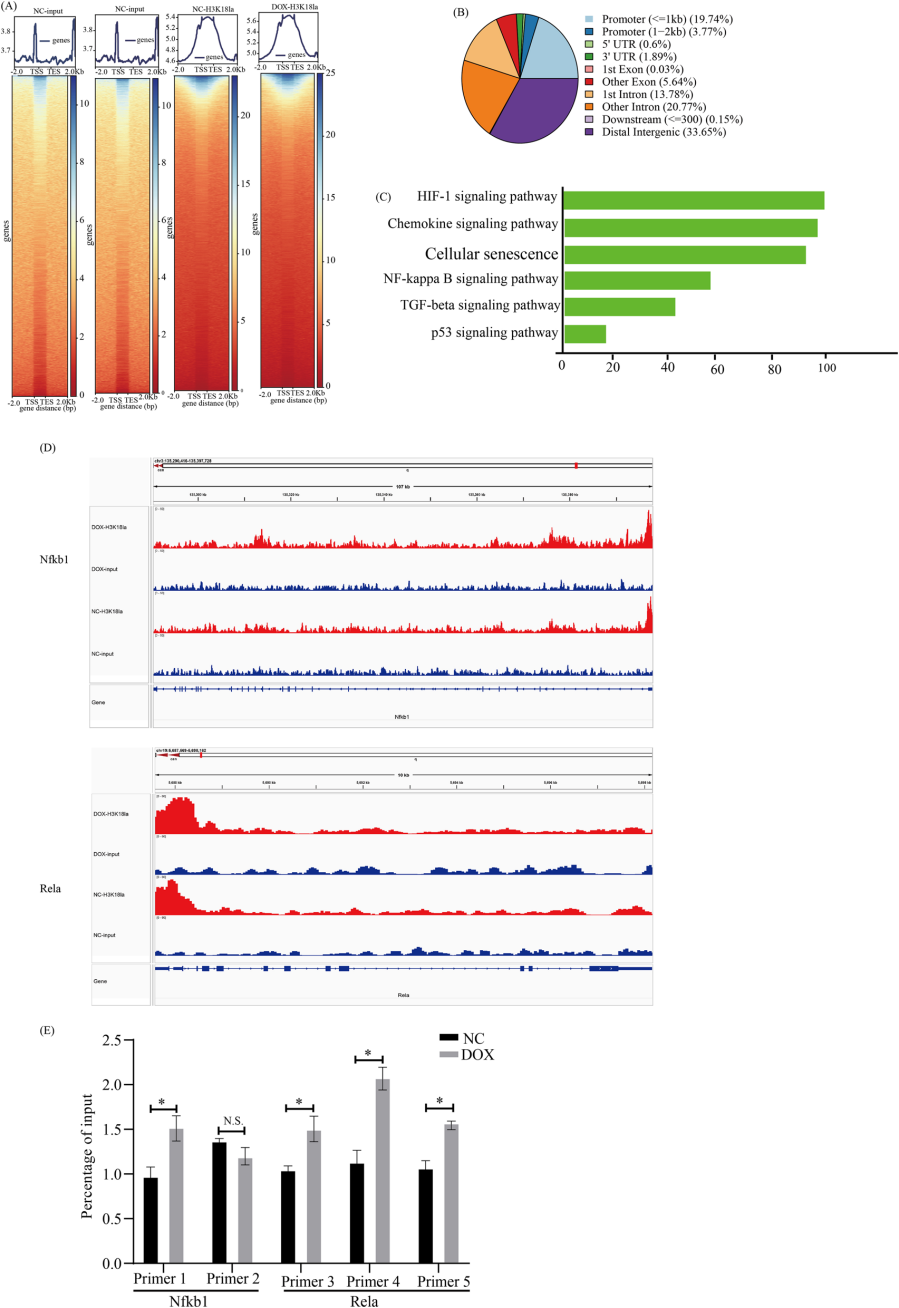
Analysis of the transcriptional consequences of H3K18la in Senescent BV2 Cells. **A** Heat map displaying the binding density of H3K18la with different H3K18la binding peaks in Dox-BV2 and NC-BV2, ordered by signal strength. **B** Genome-wide distribution of upregulated H3K18la-binding peaks in Dox-BV2. **C** KEGG analysis of enhanced H3K18la binding peaks at candidate target genes. **D** Genome browser tracks of H3K18la binding peaks at the representative target gene loci. The red rectangles indicate the peak regions of H3K18la on Nfkb1or Rela promoters. **E** Chip-qpcr analysis of the indicated promoter’s regions of Nfkb1 and Rela was performed using antibodies against H3K18la in Dox-BV2 and NC-BV2. Each bar represents the mean ± s.d. for triplicate experiments, *p < 0.05, **p < 0.01, ***p < 0.001, N.S.: no significance

Histone acylation modifications can both promote and inhibit the transcription of downstream target genes [49]. To explore the downstream target genes regulated by H3K18la and their potential biological functional significance in the process of microglia senescence, brain aging and AD, we performed chromatin immunoprecipitation sequencing (Chip-seq) to identify candidate genes regulated by H3K18la in senescent microglia. Chip-seq using antibodies against H3K18la in senescent BV2 cells (Dox-BV2) and young BV2 cells (NC-BV2) revealed obvious enrichment of H3K18la peaks in Dox-BV2 compared to NC-BV2 (Fig. 6A). The comparison between Dox-BV2 and NC-BV2 showed 21,774 differential H3K18la binding peaks, with 19.74% of the peaks located within promoter sequences (≤ 1 kb), and with 3.77% of the peaks located within promoter sequences (1 kb–2 kb) (Fig. 6B).

To investigate the potential target genes regulated by H3K18la in senescent microglia, a total of 11,796 target gene of different H3K18la binding peaks at the promoter in senescent microglia were classified into different KEGG pathways. These KEGG pathways include the chemokine signaling pathway, cellular senescence, NF-kappa B signaling pathway, TNF-signaling pathway and p53 signaling pathway, which are involved in aging and inflammation (Fig. 6C). Specifically, the peaks identified candidate genomic loci at NFκB genes, including NFκB1 (gene ID: 18033, location: chr3: 135397308–135397660) and Rela (gene ID: 19697, location: chr19: 5686901–5687340), showed that the abundance of H3K18la binding on the promoters of these genes in Dox-BV2 was significantly elevated compared to NC-BV2 (Fig. 6D).

Chip quantitative Polymerase Chain Reaction (Chip-qPCR) analysis using primer pairs (Additional file 2: Table S1) targeting potential promoter-binding regions revealed that the H3K18la levels on promoters of Rela and NFκB1 were significantly elevated in Dox-BV2 compared with Dox-BV2 (Fig. 6E). H3K18la has three binding sites on the ReLa promoter region, and one binding site on the NFΚB 1 promoter. To further validate the epigenetic role of H3K18la, we synchronously performed RNA-seq to analyze the transcriptional expression profile of Dox-BV2 and NC-BV2. Consistently, the KEGG analysis from the RNA-seq data set showed the signaling pathway enrichment of NFκB and Cytokine–cytokine receptor interaction (Additional file 1: Fig. S4). Combined with data from Chip-qPCR and RNA-seq, we conclude that H3K18la directly stimulates the NFκB signaling by enhanced binding on promoter regions of Rela and NFκB1.

### H3K18la/NFκB signaling axis promotes brain aging and Alzheimer's disease phenotype by potentiating SASP components IL-6 and IL-8

**Fig. 7:**
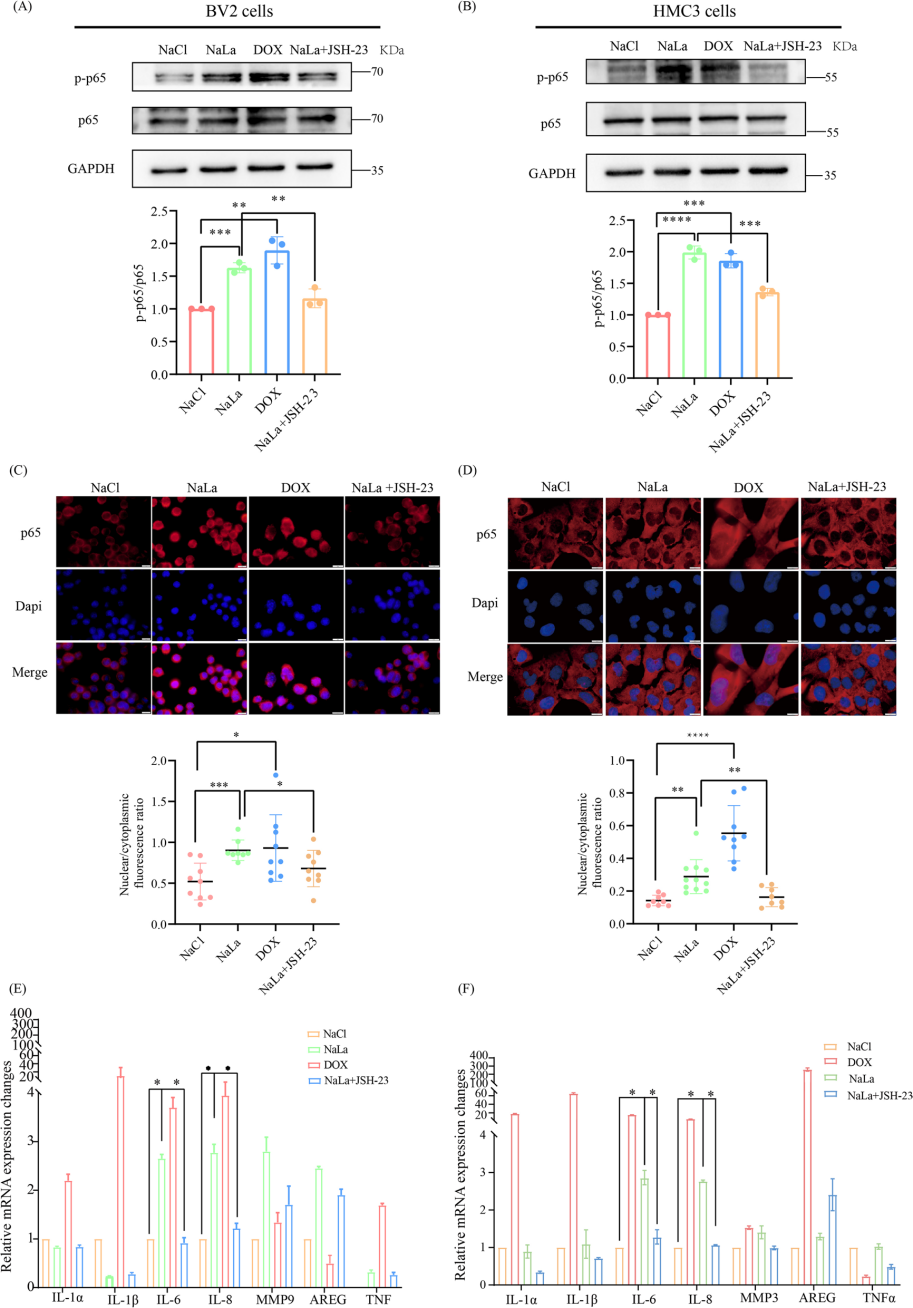
H3K18la/NFκB signaling axis aggravate brain aging and Alzheimer's disease pathology by upregulating IL-6 and IL-8. **A**, **B** Immunoblotting results displaying changes in levels of p–p65 and p65 in BV2 (**A**) or HMC3 (**B**) cells treated with indicated. **C**, **D** Cell immunofluorescence results indicating the nuclear activation of p65 in BV2 (**C**) or HMC3 (**D**) cells treated with indicated, scale bar, 10 μm. **E**, **F** q-PCR detection of the expression levels of SASP components in BV2 (**E**) or HMC3 (**F**) cells treated with indicated. Data are presented as means ± SD from three biological experiments. Each bar represents the mean ± s.d. for triplicate experiments, *p < 0.05, **p < 0.01, ***p < 0.001, N.S.: no significance

To investigate the effect of the H3K18la/NFκB signaling axis on the SASP, we treated proliferating BV2 and HMC3 cells with or without Nacl, dox, lactate or NFκB inhibitor JSH-23. First, an immunoblotting assay validated that lactate-treated proliferating BV2 and HMC3 cells led to elevated p–p65 level in relation to cells treated with Nacl, while proliferating BV2 or HMC3 cells simultaneously treated with JSH-23 reverse the increased p–p65 (Fig. 7A, B). Moreover, cell immunofluorescence also verified that lactate-treated proliferating BV2 and HMC3 cells enhanced the expression of activated p65 in nucleus compared to cells treated with NaCl, while lactate-treated proliferating BV2 and HMC3 cells simultaneously treated with JSH-23 reduced nuclear activation of p65, which is consistent with the results from the immunoblotting assay (Fig. 7C, D). Q-PCR data found that lactate-treated proliferating BV2 and HMC3 cells showed obvious increase in IL-6 and IL-8 levels compared to cells treated with NaCl, suggesting that the elevated SASP components were triggered by lactate-induced H3K18la. In contrast, lactate-treated proliferating BV2 or HMC3 cells simultaneously treated with JSH-23 reversed the H3K18la-induced increases in IL-6 and IL-8, indicating that lactate-induced enhanced H3K18la led to elevated IL-6 and IL-8 through the NFκB signaling in BV2 and HMC3 cells (Fig. 7E, F). Taken together, our data substantiate that the H3K18la/NFκB signaling axis regulates IL-6 and IL-8, which affect brain aging and Alzheimer's disease phenotype.

## Discussion

Compelling evidence suggests that abnormal histone modifications influences the translation of cellular metabolic intermediates into changes in gene transcription and expression [50]. This is mediated by cellular intermediary metabolites which serve as cofactors that either add or remove chromatin modifications, induced by chromatin modifying enzymes. Concentration changes in these cellular metabolic intermediates may up- or down-regulate gene expression by altering chromatin states [51]. A recent study found that Lactate, a product of glycolysis and a significant energy source, can regulates gene transcription via lactylation of histones through fluctuations in lactate content in cells, representing a new PTM contributor to the epigenetic landscape.

Several lines of evidence suggest that senescent cells are still metabolically active, and can induce changes in their environment through secreted molecules or by switching energy metabolism fashion [52, 53]. Senescent cells are associated with a shift towards glycolysis [54]. In this work, we found that lactate levels are significantly increased in senescent microglia, indicating that senescent microglia switch their metabolism from OXPHOS to aerobic glycolysis, which produces 5′-triphosphate (ATP) rapidly but also generates massive lactate. Moreover, senescent microglia-trigged accumulation of lactate caused enhanced histone Kla levels, contributing to the development and progression of brain aging and AD pathogenesis. These preliminary results suggest that the metabolic transition to aerobic glycolysis of senescent microglia may also affect itself and its local environment by affecting neuroinflammation through histone Kla-mediated epigenetics. In the epigenetic mechanism, our chip-Seq and chip-qPCR disclosed H3k18la stimulates the NFκB pathway to promote SASP components IL-6 and IL-8 in senescent microglia, which suggests that the metabolic transition to aerobic glycolysis of senescent microglia aggravates brain aging and AD pathology by targeting to neuroinflammation through the H3k18la/NFκB signal axis. Our data expand the biological function of histone Kla and histone Kla-regulated downstream target gene or signaling pathway. Our results preliminarily uncover that the H3K18 lactylation/NFκB signaling axis in senescent microglia can aggravate brain aging and AD phenotype by potentiating SASP, which is partly in agreement with a previous study reporting an H4K12 lactylation/PKM2 positive feedback loop in microglia that drives the pathogenesis of AD [24, 25]. Taken together, the above results suggest that different histone lactylations act through different molecular mechanics by modulating distinct target genes or pathways in microglia to influence brain aging and AD phenotype. Several lines of evidence have shown that H3K27 acetylation, H4K16 acetylation, H3K9 acetylation, H3K4 methylation, H3K79 methylation, and H3K9 are implicated in the epigenetic regulation of the SASP in a PTM manner. Thus, our study found that H3K18 lactylation epigenetically regulates the SASP through NFκB signaling, broadening the list of epigenetic regulatory members of the SASP in a novel PTM aspect.

Pan.et al. found that H4K12la and H3K18la were elevated in the hippocampus and cortex tissues of AD modeling mice. Enhanced H4K12la aggravated the cognitive impairment phenotype through the glycolysis/H4K12la/PKM2 signaling axis [24]. In contrast, we uncovered that H3K18la is significantly increased in the hippocampus tissues of naturally aged and AD mice, but remained unchanged in the cortex tissues of naturally aged and AD modeling mice. This indicates that H4K12la and H3K18la are expressed in different localizations, have distinct biological functions, and downstream targets during the pathogenesis of AD. H4K12LA promotes AD pathology by regulating key glycolytic enzyme genes (such as PKM2), while H3K18la promotes AD pathology by regulating inflammation-related signaling pathways (such as NFκB).

In the present study, we confirm that the elevated lactate levels extensively exist in senescent microglial, and hippocampus tissues of naturally aged mice and AD mice, driving pan-lactylation and histone lactylation by histone lysine lactylases. Furthermore, we present the landscape of lactylome in BV2 cells by MS, and validate that H3K18la is substantially elevated in hippocampus tissues of naturally aged mice and AD mice among the MS-generated histone lactylation sites. Mechanically, we found that enhanced H3K18la leads to enhanced binding on the promoter region of Rela and NfκB1, thereby potentiating NFκB signaling and ultimately promoting SASP components IL-6 and IL-8, which dramatically impact brain aging and AD pathology. Our study suggests that there is an H3K18la/NFκB axis/SASP positive feedback loop driving the pathogenesis of brain aging and AD. Furthermore, we also found that the acetyltransferase p300/CBP and PCAF is the histone lactylases (also termed “writer”) both in 293T, Hela, BV2 and HMC3 C cells (Additional file 1: Fig. S5), which is partly in line with previous study by Zhang et al. [32] and Moreno-Yruela et al. who identified class I histone deacetylases (HDAC1-3) as histone lysine delactylases (also termed “eraser”) [55].

**Fig. 8:**
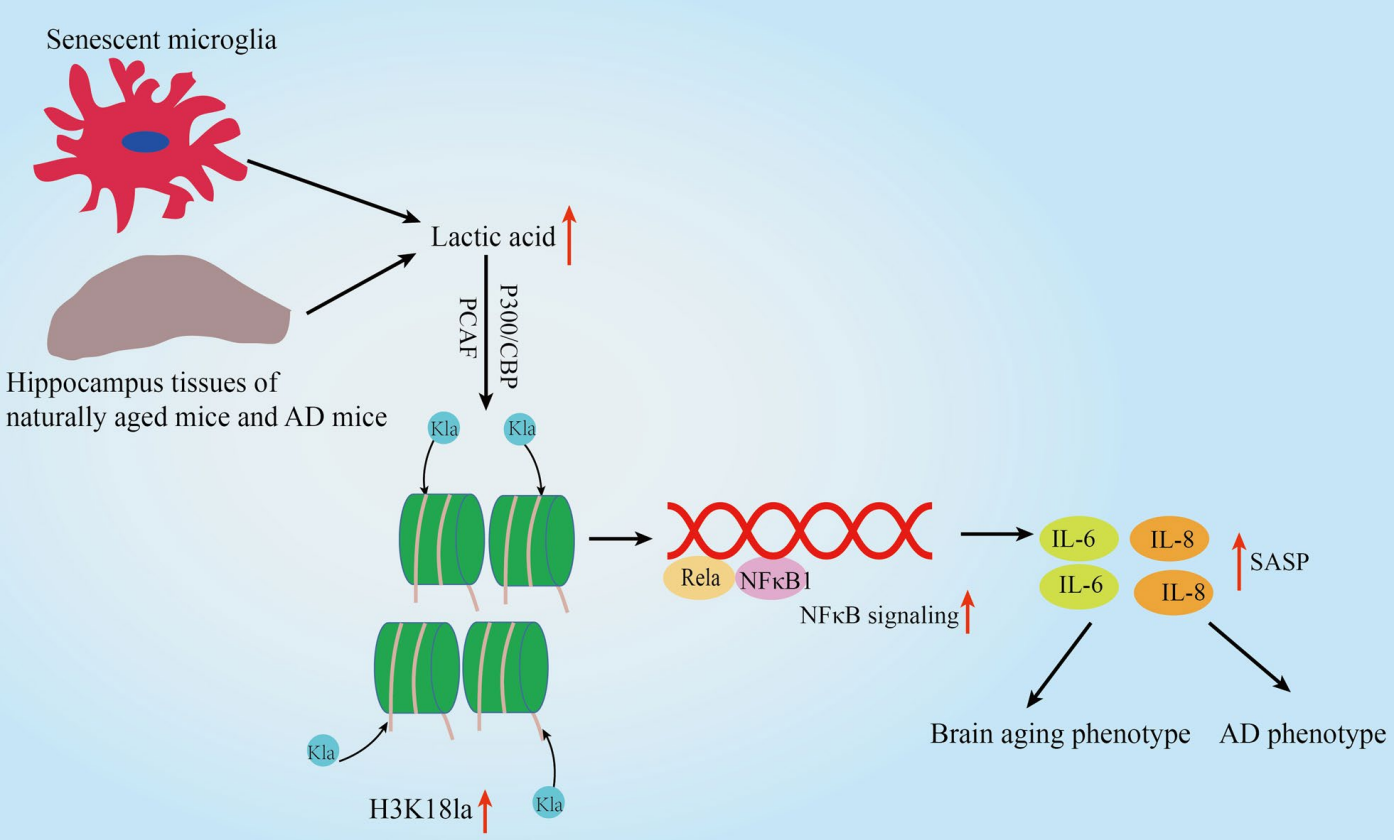
Proposed working model illustrating the lactate/H3K18la/NFκB axis in senescent microglial, and hippocampus tissues of naturally aged mice and AD mice driving the pathogenesis of brain aging and AD. Increased lactate levels in senescent microglial, and hippocampus tissues of naturally aged mice and AD mice increases H3K18la via histone lysine lactylases CBP/P300 and PCAF. Enhanced H3K18la stimulates IL-6 and IL-8 by enhancing NFκB signaling, which affects brain aging and AD pathology

Collectively, these findings showed a novel molecular mechanism in which H3K18la/NFκB axis modulates inflammation associated with aging (infammageing) by regulating SASP components IL-6 and IL-8, promoting brain aging and AD pathological phenotypes (Fig. 8).These results present potential targets for the development of drug interventions for brain aging and AD pathology.

### Supplementary Information

**Additional file 1: Figure S1.** Identification of senescent microglia and hippocampus tissues of naturally aged mice. **A**, **B** Immunoblotting detection of the expression level of p53 and p21 in the indicated BV2 cells (**A**) and HMC3 cells (**B**). **C, D** Proliferative capacity of BV2_NC, BV2_Dox, HMC3_NC and HMC3_ Dox as determined by an RTCA SP system after 120 h, the blue arrows indicate the timepoint of doxorubicin addition. **E**, **F** Senescence-associated beta-galactosidase (SA-β-gal) staining of BV2_NC, BV2_Dox, HMC3_NC and HMC3_ Dox. **G**, **H** Clonogenicity assay of BV2_NC, BV2_Dox, HMC3_NC and HMC3_ Dox. **I** Immunoblotting detection of the expression level of p53 and p16 in hippocampus tissues of young and naturally aged mice (*n* = 4 mice per group). **J** SA-β-gal staining of hippocampus tissues of young and naturally aged mice (*n* = 4 mice per group), scale bar, 50 μm. Each bar represents the mean ± s.d. for triplicate experiments, *p < 0.05, **p < 0.01, ***p < 0.001. All experiments were performed as three independent biological replicates. **Figure S2.** Confirmation of the non-histone Pan-Kla level in senescent microglia, hippocampus, and cortex of naturally aged mice. **A** Immunoblotting analyzes of non-histone Pan-Kla level in the indicated BV2 cells (left plot) and HMC3 cells (right plot). **B**, **C** Immunoblotting analysis of non-histone Pan-Kla level in hippocampus (**B**) and cortex (**C**) of naturally aged mice (*n* = 4 mice per group). **Figure S3. A** Description of the Kla structure. **B** Representative images of MS/MS spectra of histone Kla. Illustration of histone Kla sites identified in human HeLa and mouse BV2 cells. **Figure S4.** TOP 20 KEGG analysis of RNA-seq data set. **A** TOP 20 KEGG pathways. **B** ScatterPlot of the Top 20 KEGG pathways. **Figure S5.** Identification of histone lysine lactylases. **A, B** Immunoblotting analysis for histone Kla alterations in 293T cells (**A**), Hela cells (**B**), BV2 cells (**C**) and HMC3 (**D**) cells transfected with vector, p300, CBP, PCAF, TP60 and hMOF, respectively. Each bar represents the mean ± s.d. for triplicate experiments, *p < 0.05, **p < 0.01, ***p < 0.001, N.S., no significance.

**Additional file 2: Table S1.** Primers used in this study.

## Data Availability

The data that support the findings of this study are available from the corresponding author upon reasonable request.
