# Supplementary material for: H3K18 lactylation of senescent microglia potentiates brain aging and Alzheimer's disease through the NFκB signaling pathway
Source: J Neuroinflammation. 2023 Sep 11;20:208. doi: 10.1186/s12974-023-02879-7 (PMC10494370; doi:10.1186/s12974-023-02879-7)
Supplement: Supplementary file 1 — Additional file 1: Figure S1. Identification of senescent microglia and hippocampus tissues of naturally aged mice. A, B Immunoblotting detection of the expression level of p53 and p21 in the indicated BV2 cells (A) and HMC3 cells (B). C, D Proliferative capacity of BV2_NC, BV2_Dox, HMC3_NC and HMC3_ Dox as determined by an RTCA SP system after 120 h, the blue arrows indicate the timepoint of doxorubicin addition. E, F Senescence-associated beta-galactosidase (SA-β-gal) staining of BV2_NC, BV2_Dox, HMC3_NC and HMC3_ Dox. G, H Clonogenicity assay of BV2_NC, BV2_Dox, HMC3_NC and HMC3_ Dox. I Immunoblotting detection of the expression level of p53 and p16 in hippocampus tissues of young and naturally aged mice (n = 4 mice per group). J SA-β-gal staining of hippocampus tissues of young and naturally aged mice (n = 4 mice per group), scale bar, 50 μm. Each bar represents the mean ± s.d. for triplicate experiments, *p < 0.05, **p < 0.01, ***p < 0.001. All experiments were performed as three independent biological replicates. Figure S2. Confirmation of the non-histone Pan-Kla level in senescent microglia, hippocampus, and cortex of naturally aged mice. A Immunoblotting analyzes of non-histone Pan-Kla level in the indicated BV2 cells (left plot) and HMC3 cells (right plot). B, C Immunoblotting analysis of non-histone Pan-Kla level in hippocampus (B) and cortex (C) of naturally aged mice (n = 4 mice per group). Figure S3. A Description of the Kla structure. B Representative images of MS/MS spectra of histone Kla. Illustration of histone Kla sites identified in human HeLa and mouse BV2 cells. Figure S4. TOP 20 KEGG analysis of RNA-seq data set. A TOP 20 KEGG pathways. B ScatterPlot of the Top 20 KEGG pathways. Figure S5. Identification of histone lysine lactylases. A, B Immunoblotting analysis for histone Kla alterations in 293T cells (A), Hela cells (B), BV2 cells (C) and HMC3 (D) cells transfected with vector, p300, CBP, PCAF, TP60 and hMOF, respectively. Each bar represent [file 12974_2023_2879_MOESM1_ESM.docx]

Additional file

H3K18 lactylation of senescent microglia potentiates brain aging and Alzheimer's disease through the NFκB signaling pathway

Lin Wei, Xiaoweng Yang, Jie Wang, Zhixiao Wang, Qiguang Wang, Yan Ding*, and Aiqing Yu*


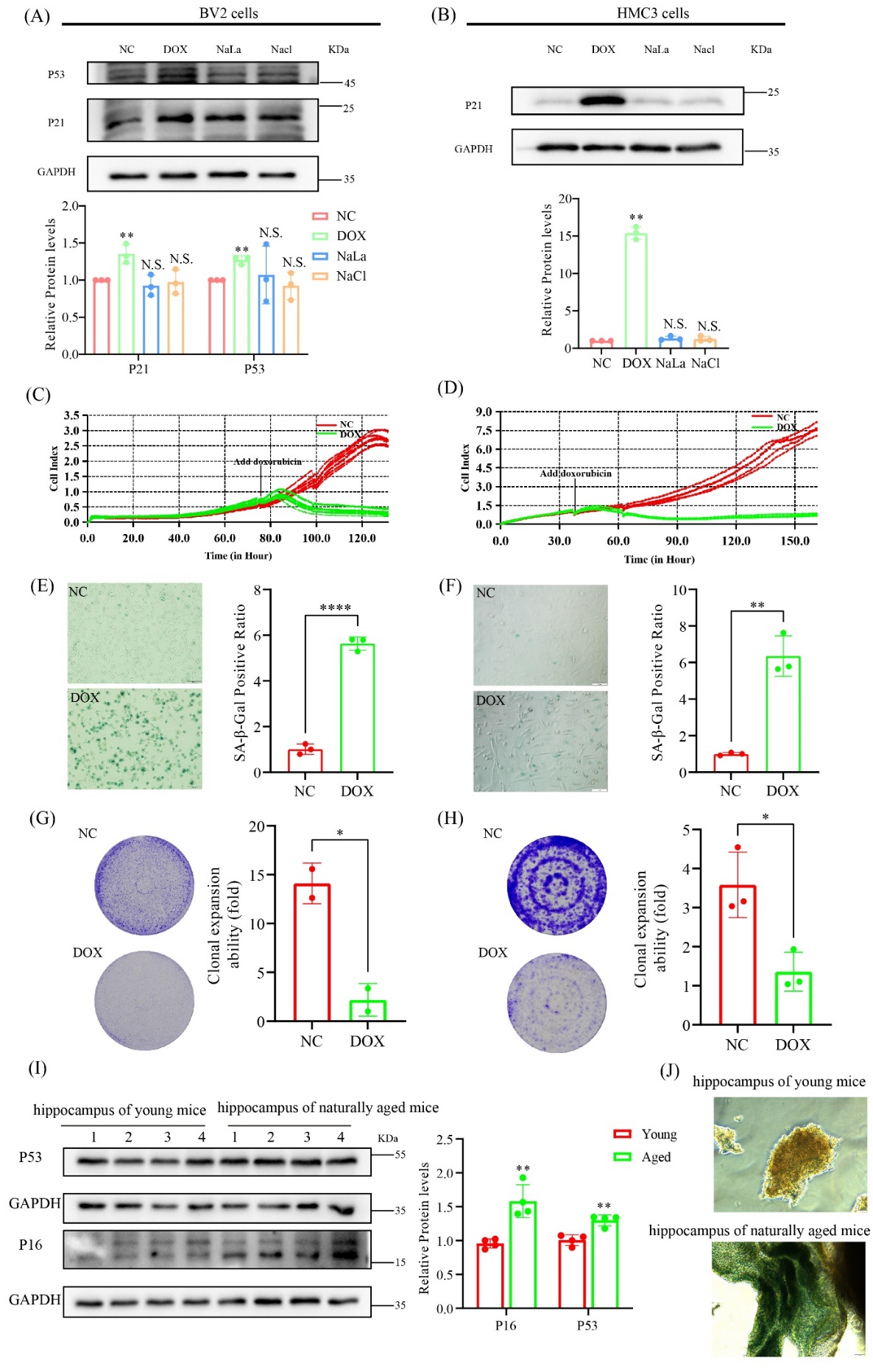


**Figure S1. Identification of senescent microglia and hippocampus tissues of naturally aged mice. A-B** Immunoblotting detection of the expression level of p53 and p21 in the indicated BV2 cells (**A**) and HMC3 cells (**B**). **C-D** The proliferative capacity of BV2_NC, BV2_Dox, HMC3_NC and HMC3_ Dox as determined by an RTCA SP system after 120 h, the blue arrows indicate the time-point of doxorubicin addition. **E-F** Senescence-associated beta-galactosidase (SA-β-gal) staining of BV2_NC, BV2_Dox, HMC3_NC and HMC3_ Dox. **G-H** Clonogenicity assay of BV2_NC, BV2_Dox, HMC3_NC and HMC3_ Dox. **I** Immunoblotting detection of the expression level of p53 and p16 in hippocampus tissues of young and naturally aged mice (n=4 mice per group). **J** SA-β-gal staining of hippocampus tissues of young and naturally aged mice (n=4 mice per group), scale bar,50 μm. Each bar represents the mean±s.d. for triplicate experiments, **p* < 0.05, ***p* < 0.01, ****p* < 0.001. All experiments were performed as three independent biological replicates.


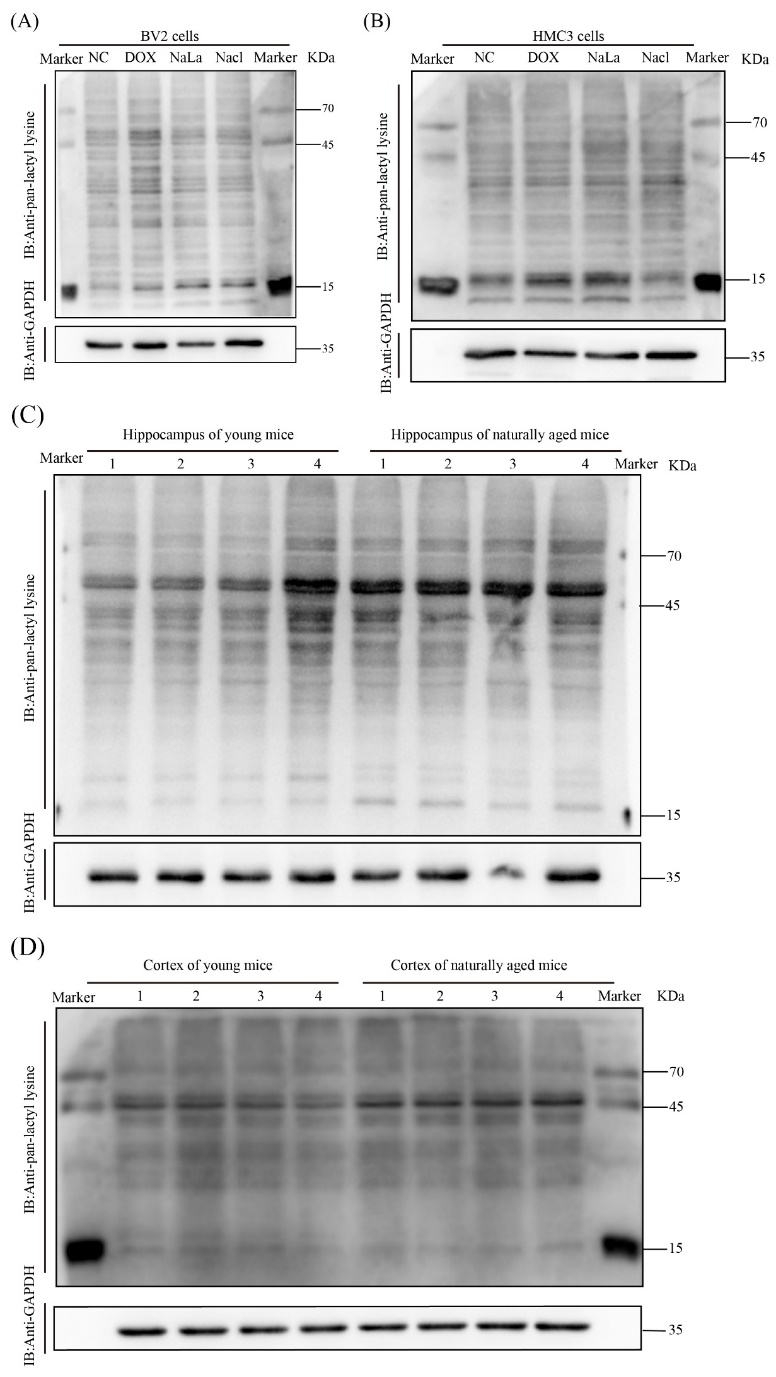


**Figure S2. Confirmation of the non-histone Pan-Kla level in senescent microglia, hippocampus, and cortex of naturally aged mice. A** Immunoblotting analyzes of non-histone Pan-Kla level in the indicated BV2 cells (left plot) and HMC3 cells (right plot). **B-C** Immunoblotting analysis of non-histone Pan-Kla level in hippocampus(**B**) and cortex(**C**) of naturally aged mice (n=4 mice per group).


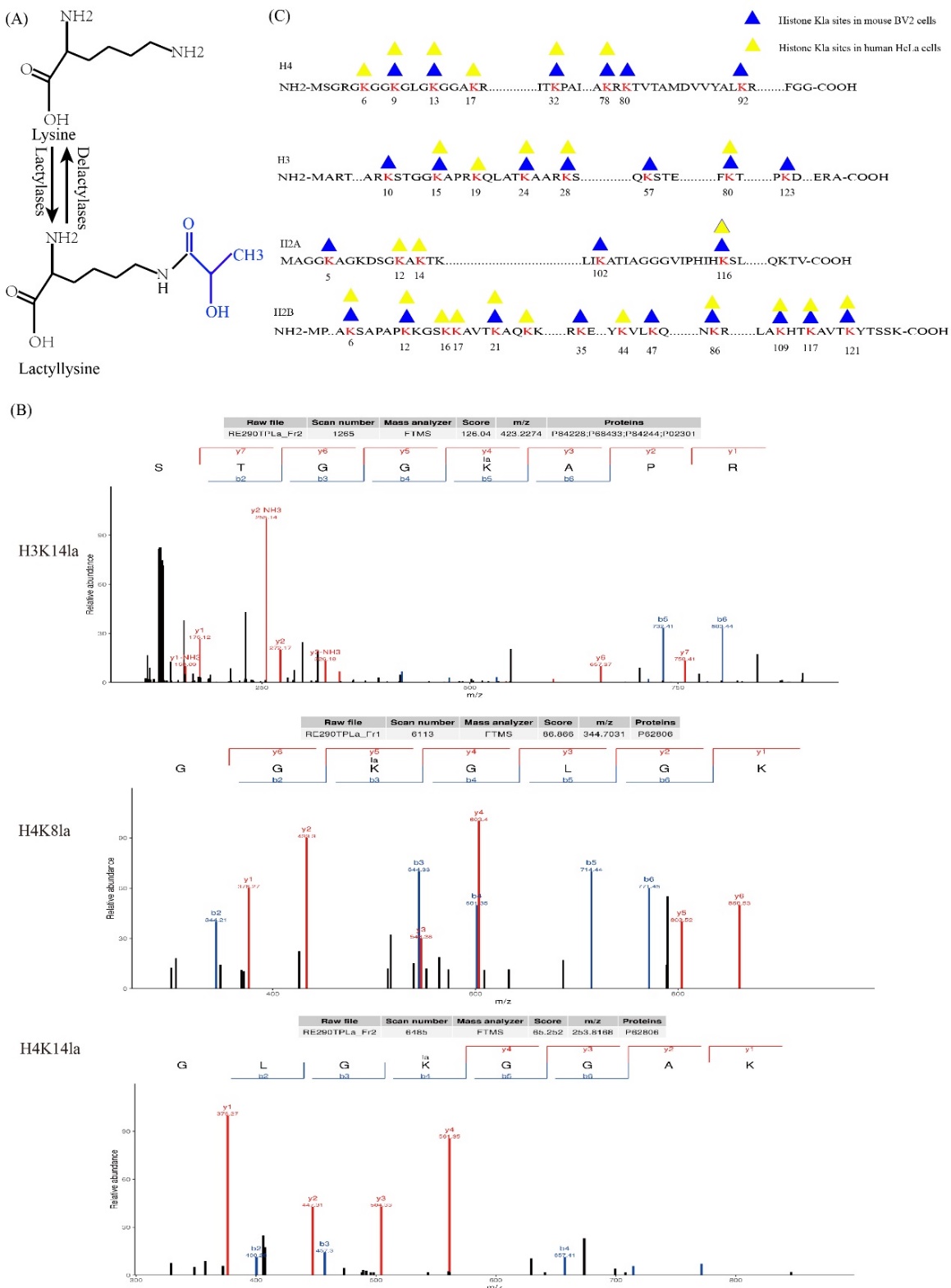


**Figure S3. A** Description of the Kla structure. **B** Representative images of MS/MS spectra of histone Kla. Illustration of histone Kla sites identified in human HeLa and mouse BV2 cells.


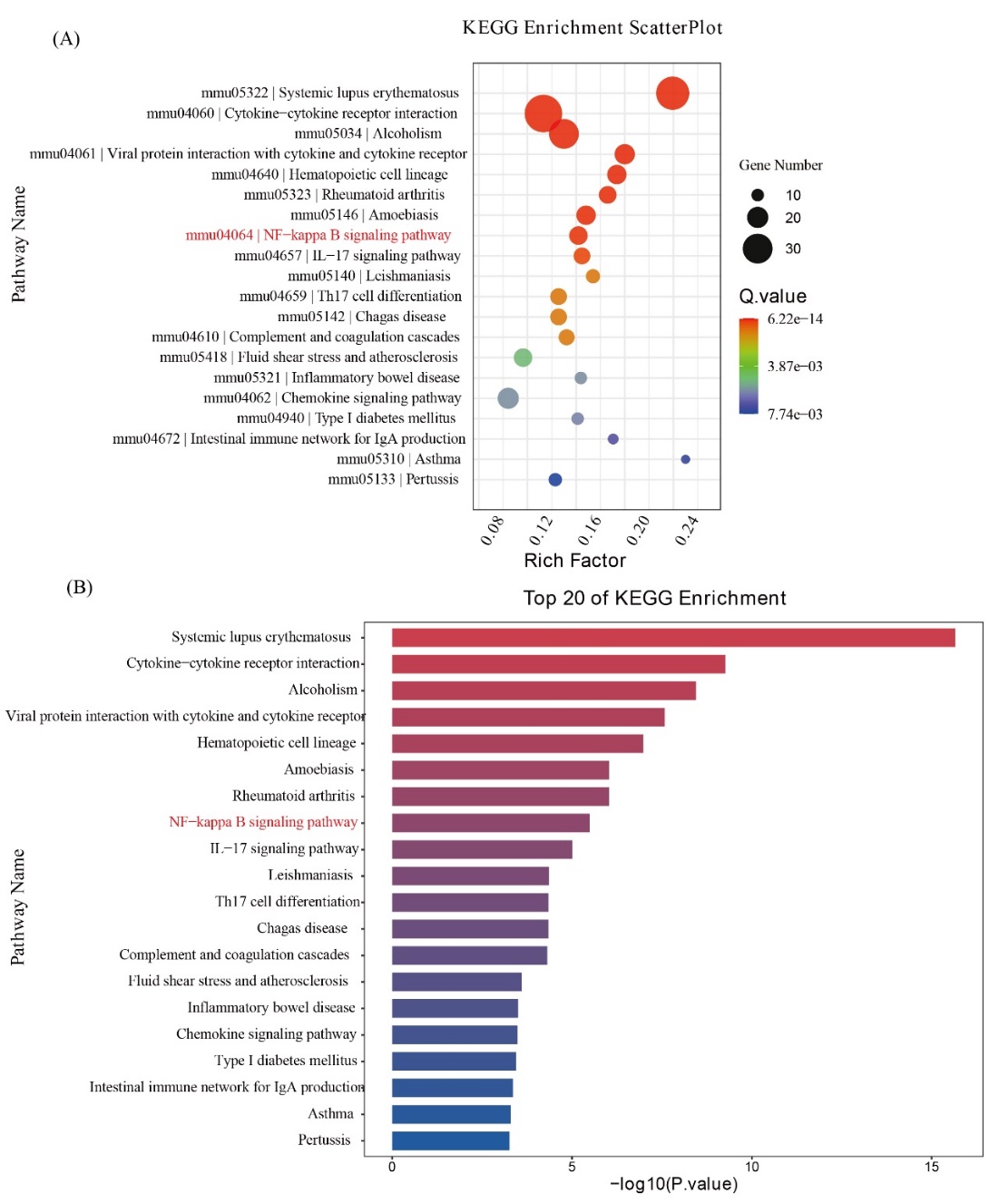


**Figure S4 The TOP 20 KEGG analysis of RNA-seq dataset. A** The TOP 20 KEGG pathways. **B** ScatterPlot of the Top 20 KEGG pathways.


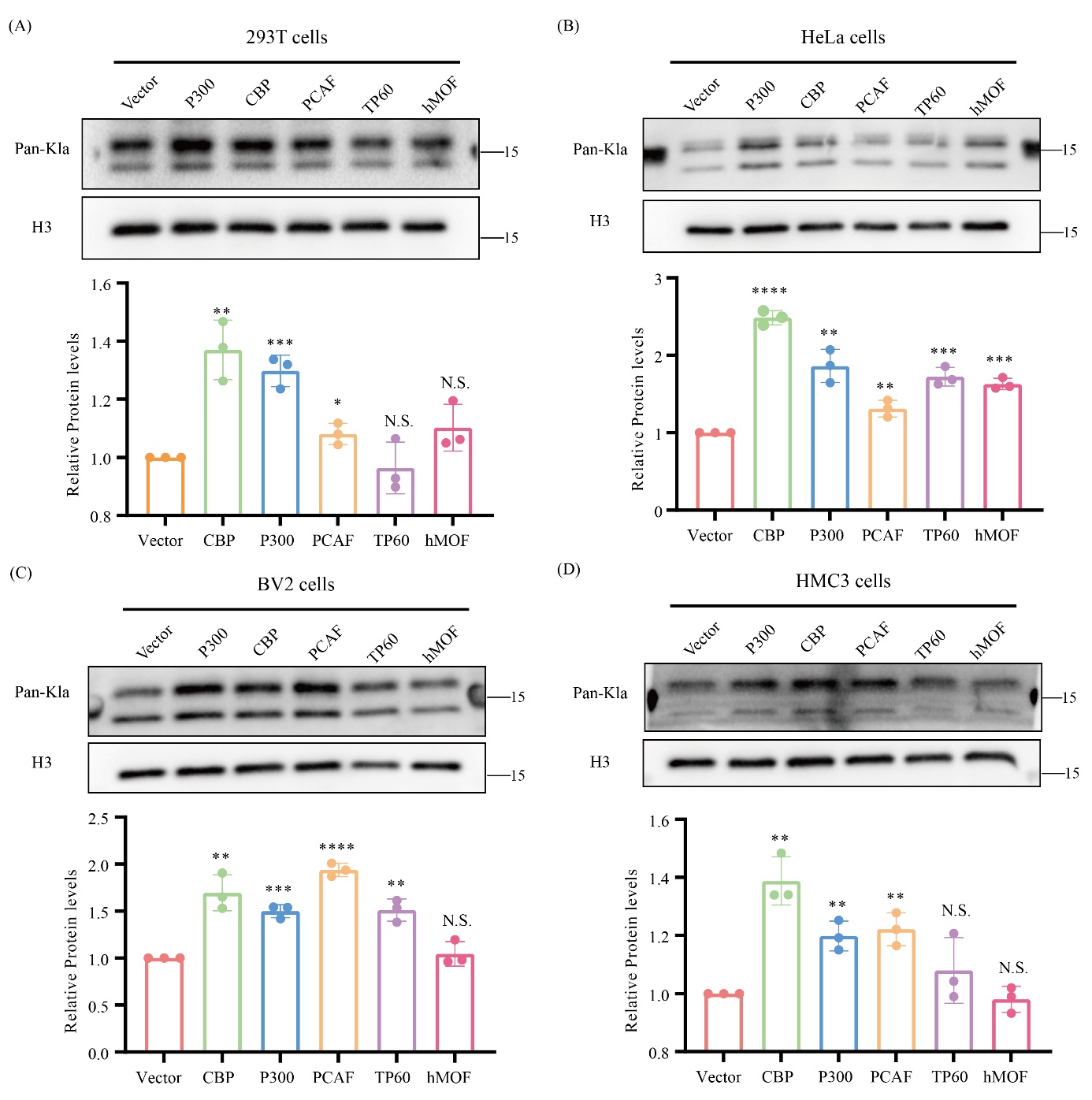


**Figure S5. Identification of histone lysine lactylases. A-B** Immunoblotting analysis for histone Kla alterations in 293T cells(**A**), Hela cells(**B**), BV2 cells (C)and HMC3(D) cells transfected with vector, p300, CBP, PCAF, TP60 and hMOF respectively. Each bar represents the mean±s.d.for triplicate experiments, *p < 0.05, **p < 0.01, ***p < 0.001, N.S., no significance.
