# Supplementary material for: H3K18 lactylation of senescent microglia potentiates brain aging and Alzheimer's disease through the NFκB signaling pathway
Source: J Neuroinflammation. 2023 Sep 11;20:208. doi: 10.1186/s12974-023-02879-7 (PMC10494370; doi:10.1186/s12974-023-02879-7)
Supplement: Supplementary file 2 — Additional file 2: Table S1. Primers used in this study. [file 12974_2023_2879_MOESM2_ESM.docx]

**Table S1** Primers used in this study

| Oligonucleotide sequences(Forward, R:Reverse) for Chip-qpcr | 5'--3' |
| --- | --- |
| p65-F1(mouse, primer 3) | GCACACCTGCCCTTGGCTTAGATG |
| p65-R1(mouse, primer 3) | CCTGAGAGCCATGAGTGCTGGAAG |
| p65-F2(mouse, primer 4) | GCGCAGCCGGATCTAGGTT |
| p65-R2(mouse, primer 4) | TCACTCTGTTTTTAGGGGATTTCGG |
| p65-F3(mouse, primer 5) | AAACCTTAGCAGACAGCACCTA |
| p65-R3(mouse, primer 5) | GAGGACTAGGCAGACACCTGT |
| Nfkb1-F1(mouse, primer 1) | AAGACTTCATCCAGACCCAA |
| Nfkb1-R1(mouse, primer 1) | TCTGCTTGTTCCCTCCTCT |
| Nfkb1-F2(mouse, primer 2) | ATGGAGCGATCTGAGTGTAG |
| Nfkb1-R2(mouse, primer 2) | GCCCCGAACCCCAAG |
| Oligonucleotide sequences(Forward, R:Reverse) for qRT-PCR | 5'--3' |
| IL-1β-F(mouse/human) | CACTACAGGCTCCGAGATGAACAAC/CTCTCCACCTCCAGGGACAGG |
| IL-1β-R(mouse/human) | TGTCGTTGCTTGGTTCTCCTTGTAC/TCAACACGCAGGACAGGTACAG |
| IL-6-F(mouse/human) | TAGTCCTTCCTACCCCAATTTCC/TGGTGTTGCCTGCTGCCTTC |
| IL-6-R(mouse/human) | TTGGTCCTTAGCCACTCCTTC/GCTGAGATGCCGTCGAGGATG |
| IL-8-F(mouse/human) | CAAGGCTGGTCCATGCTCC/ACCACACTGCGCCAACACAG |
| IL-8-R(mouse/human) | TGCTATCACTTCCTTTCTGTTGC/AACCCTCTGCACCCAGTTTTCC |
| IL-1α-F(mouse/human) | GCACCTTACACCTACCAGAGT/ACCAACCAGTGCTGCTGAAGG |
| IL-1β-R(mouse/human) | AAACTTCTGCCTGACGAGCTT/AGTGCCGTGAGTTTCCCAGAAG |
| MMP3-F(mouse/human) | ACATGGAGACTTTGTCCCTTTTG/GATTGGAGGTGACGGGGAAGC |
| MMP3-R(mouse/human) | TTGGCTGAGTGGTAGAGTCCC/TTCGGGATGCCAGGAAAGGTTC |
| AREG-F(mouse/human) | GAAGACTCACAGCGAGGATGACAAG/CGCTCTTGATACTCGGCTCAGG |
| AREG-R(mouse/human) | TGATAACGATGCCGATGCCAATAGC/GGTTCACGCTTCCCAGAGTAGG |
